# Contraceptive Use and the Risk of Ectopic Pregnancy: A Multi-Center Case-Control Study

**DOI:** 10.1371/journal.pone.0115031

**Published:** 2014-12-10

**Authors:** Cheng Li, Wei-Hong Zhao, Chun-Xia Meng, Hua Ping, Guo-Juan Qin, Shu-Jun Cao, Xiaowei Xi, Qian Zhu, Xiao-Cui Li, Jian Zhang

**Affiliations:** 1 Department of Obstetrics and Gynecology, International Peace Maternity and Child Health Hospital, School of Medicine, Shanghai Jiaotong University, Shanghai, 200030, China; 2 Department of Obstetrics, Gynecology and Women’s Health, School of Medicine, University of Missouri-Columbia, Columbia, MO, 65212, United States of America; 3 Department of Obstetrics and Gynecology, Songjiang Maternity and Child Health Hospital, Shanghai, 201620, China; 4 Department of Obstetrics and Gynecology, Minhang Central Hospital, Shanghai, 201100, China; 5 Department of Obstetrics and Gynecology, Songjiang Central Hospital, Shanghai, 201600, China; 6 Department of Obstetrics and Gynecology, Shanghai First People’s Hospital, Shanghai Jiaotong University, Shanghai, 200080, China; State University of Maringá/Universidade Estadual de Maringá, Brazil

## Abstract

**Objective:**

To evaluate the association between the risk of ectopic pregnancy (EP) and the use of common contraceptives during the previous and current conception/menstrual cycle.

**Methods:**

A multi-center case-control study was conducted in Shanghai. Women diagnosed with EP were recruited as the case group (n = 2,411). Women with intrauterine pregnancy (IUP) (n = 2,416) and non-pregnant women (n = 2,419) were matched as controls at a ratio of 1∶1. Information regarding the previous and current use of contraceptives was collected. Multivariate logistic regression analyses were performed to calculate odds ratios (ORs) and the corresponding 95% confidential intervals (CIs).

**Results:**

Previous use of intrauterine devices (IUDs) was associated with a slight risk of ectopic pregnancy (AOR_1_ = 1.87 [95% CI: 1.48–2.37]; AOR_2_ = 1.84 [1.49–2.27]), and the risk increased with the duration of previous use (*P_1_* for trend <10^−4^, *P_2_* for trend <10^−4^). The current use of most contraceptives reduced the risk of both unwanted IUP (condom: AOR = 0.04 [0.03–0.05]; withdrawal method: AOR = 0.10 [0.07–0.13]; calendar rhythm method: AOR = 0.54 [0.40–0.73]; oral contraceptive pills [OCPs]: AOR = 0.03 [0.02–0.08]; levonorgestrel emergency contraception [LNG-EC]: AOR = 0.22 [0.16–0.30]; IUDs: AOR = 0.01 [0.005–0.012]; tubal sterilization: AOR = 0.01 [0.001–0.022]) and unwanted EP (condom: AOR_1_ = 0.05 [0.04–0.06]; withdrawal method: AOR_1_ = 0.13 [0.09–0.19]; calendar rhythm method: AOR_1_ = 0.66 [0.48–0.91]; OCPs: AOR_1_ = 0.14 [0.07–0.26]; IUDs: AOR_1_ = 0.17 [0.13–0.22]; tubal sterilization: AOR_1_ = 0.04 [0.02–0.08]). However, when contraception failed and pregnancy occurred, current use of OCPs (AOR_2_ = 4.06 [1.64–10.07]), LNG-EC (AOR_2_ = 4.87 [3.88–6.10]), IUDs (AOR_2_ = 21.08 [13.44–33.07]), and tubal sterilization (AOR_2_ = 7.68 [1.69–34.80]) increased the risk of EP compared with the non-use of contraceptives.

**Conclusion:**

Current use of most contraceptives reduce the risk of both IUP and EP. However, if the contraceptive method fails, the proportions of EP may be higher than those of non-users. In the case of contraceptive failure in the current cycle, EP cases should be differentiated according to current use of OCPs, LNG-EC, IUDs, and tubal sterilization. In addition, attention should be paid to women with previous long-term use of IUDs.

## Introduction

Ectopic pregnancy (EP) is a major cause of maternal morbidity and, occasionally, mortality. Better understanding of the risk factors for EP can aid in early diagnosis and avoid potentially life-threatening emergencies and the resulting physical and psychological harm to women [1]. In recent decades, the incidence of EP has increased [2]. Contraceptive failure has been considered one of the important factors associated with this increased EP incidence [3].

The use of long-term contraceptives, including intrauterine devices (IUDs), oral contraceptive pills (OCPs), and tubal sterilization, and short-term contraceptive methods, such as condoms, rhythm methods, withdrawal, and levonorgestrel emergency contraception (LNG-EC), are the most commonly used methods in China [4]. All of these contraceptives, whether hormonal or mechanical, can effectively protect women from unintended pregnancy. However, any form of contraceptive has a certain probability of failure, which can lead to an unexpected pregnancy, including an EP. One study revealed that contraceptive devices may be less effective for ectopic pregnancy prevention than for intrauterine pregnancy prevention, meaning that pregnancies resulting from contraceptive failure may be more likely to be ectopic [2]. The risk of EP resulting from contraceptive failure varies according to the contraceptive method used [5].

In 1995, Parazzini *et al.* conducted a case-control study on the relationship between the past use of contraceptives and the risk of EP [6]. However, with the more recent improvements in various contraceptive methods, including IUDs and OCPs, it remains unknown whether the association between these methods and the risk of EP is consistent with that reported decades ago. Furthermore, although novel methods, such as LNG-EC, have been widely used because of their high efficiency and good tolerance, cases of EP following LNG-EC failure have been consistently reported [7]–[12]. It remains unclear whether LNG-EC failure increases the risk of EP. Since the 1990s, attitudes toward different methods have changed [13]–[15], as has the overall prevalence of contraceptive use in China [4], [16]. Therefore, we were interested in the association between the risk of EP and the use of common contraceptives after the patterns of contraceptive use changed. Additionally, neither the study by Parazzini *et al.* nor other epidemiology studies has reported the association between EP risk and current use of various contraceptives since 1995. For the abovementioned reasons, we designed this case-control study and conducted it in five medical hospitals in Shanghai to re-evaluate the relationship between EP risk and common contraceptive methods used during the previous and current menstrual cycle. According to Weiss *et al.*, the odds ratio (OR) of the relationship between contraceptives and EP risk in case-control studies can vary depending on the composition of the control group [17]. Thus, a group of women with intrauterine pregnancy (IUP) and a group of non-pregnant women were recruited to constitute the control group.

## Materials and Methods

### Study design and participants

This case-control study was conducted from March 2011 through April 2013 at five medical hospitals in Shanghai (two general hospitals and three maternity hospitals). The objective of this study was to evaluate the association between EP risk and the use of different contraceptives in the previous conception cycle and the association between EP and contraceptive use in the current conception cycle using women with IUPs and non-pregnant women as controls.

The cases and controls (including both IUP and non-pregnant controls) were women of reproductive age (17 to 45 years of age), with regular sexual activity and no history of vascular disease, epilepsy, cancer, or any other diseases that could have influenced their choice of contraceptive method. The use of contraceptives such as spermicides, subcutaneous implant devices, and mifepristone for emergency contraception (this drug is legal in China) was beyond the scope of this study because they are not widely used in Shanghai. Women with a diagnosed EP (based on the American College of Obstetricians and Gynecologists [ACOG] Practice Bulletin [18]) who were seen in the inpatient department of gynecology of each hospital were interviewed as potential EP subjects. The IUP controls originated from the prenatal clinic and family planning clinic of the same hospital and were matched with the study group for age (±5 years), marital status (married or unmarried), and gestational age (±7 days) in a 1∶1 ratio. The non-pregnant control subjects were recruited from the physical examination center of each hospital and were matched with the study group for age (±5 years) and marital status (married or unmarried) in a 1∶1 ratio.

### Definition of previous and current contraceptive use

**Figure 1:**
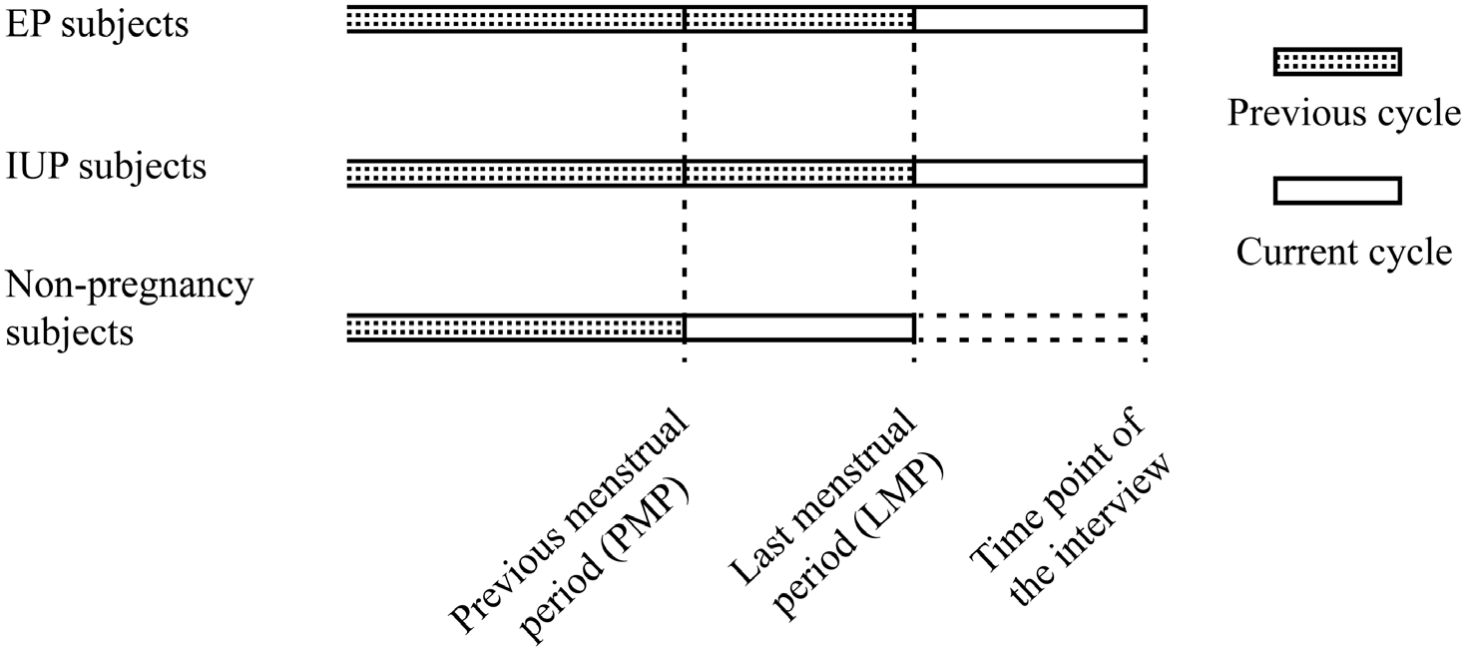
The definition of previous cycle and current cycle.

The definitions of previous and current cycles are shown in Fig. 1. A woman was considered a user of a given contraceptive method if she had used a short-term contraceptive method, including condoms, withdrawal, the calendar rhythm method, or ECP at least once or had used a long-term method, such as an IUD, OCP, or tubal sterilization, for at least one menstrual cycle. A woman was defined as a previous user of a given contraceptive method if she had used the method in the previous cycle and as a current user if she had used the method in the current cycle. Women who had undergone tubal sterilization were considered current users, and those who had undergone reversal of tubal sterilization were considered previous users. A woman was considered a previous IUD user if she had an IUD inserted that was later removed; current IUD users were those who had an IUD in situ at the time of the interview. Women were considered current non-users of contraception if they had not used any contraceptive method during the current cycle.

### Data and sample collection

The subjects were interviewed in person using a structured questionnaire to obtain information on sociodemographic characteristics (including age, marital status, birthplace, education attainment, occupation, personal annual income, and tobacco use), history of reproduction and gynecological disease (including number of previous abortions, parity, history of previous EP, and previous infertility), previous surgery (including a history of cesarean section, adnexal surgery, and appendectomy), previous contraceptive experience (including previous use of condoms, withdrawal method, calendar rhythm method, OCPs, LNG-EC, tubal sterilization, and IUD and the duration of use), and the contraceptive method used in the current conception cycle. The subjects were allowed to skip the queries if they were reluctant to reply. Queries to which the subjects did not reply were considered missing.

Five-milliliter blood samples were collected from each subject to detect the serum *Chlamydia trachomatis* (CT) IgG antibody level using an enzyme-linked immunosorbent assay (ELISA; Beijing Biosynthesis Biotechnology, China) according to the manufacturer’s instructions.

### Statistical analysis

Pearson’s chi-square tests were conducted to detect the differences between the three groups with regard to sociodemographic characteristics and previous surgery. The odds ratios (ORs) and the corresponding 95% confidence intervals (CIs) were calculated to estimate the relationship between the EP risk and different contraceptive methods (previously and currently used) and were also adjusted for potential confounding factors in the multivariate logistic regression analyses.

During multivariate analysis of the association between previous use of contraceptive methods and the current EP occurrence, we adjusted for the following potential confounding factors: birthplace (Shanghai or outside Shanghai), education attainment (college or above, high school, middle school, or primary school or lower), occupation (employed, self-employed, or unemployed), parity (0, 1, or more than 2), previous EP (no or yes), CT IgG antibody test (negative or positive), previous infertility (no or yes), previous adnexal surgery (no or yes), and previous appendectomy (no or yes). When analyzing the association between current contraceptive choice and EP risk, ORs and the corresponding 95% CIs were adjusted for all of the confounding factors described above and for the previous use of contraceptives, including condoms, withdrawal method, calendar rhythm method, OCPs, LNG-EC, tubal sterilization, and IUD (no or yes).

Tests for trend were performed by entering categorical variables into the regression model as continuous variables to detect their trend association with EP occurrence.

All statistical analyses were performed using SAS software, version 8.2 (SAS Institute, Inc., Cary, NC). All *p*-values were calculated using two-sided tests and were considered statistically significant if *p* was less than 0.05.

### Ethical considerations

This study was approved by the institutional review boards of all five hospitals (including International Peace Maternity and Child Health Hospital, Shanghai First People’s Hospital, Songjiang Central Hospital, Songjiang Maternity and Child Health Hospital, Minhang Central Hospital). All of the participants were informed about the objective of the study, and written informed consent was obtained before recruitment; for adolescent subjects younger than 18 years, written consent was obtained from their guardians. The participants were informed of their right to refuse the interview and to withdraw from the study at any time, and they were told that their information would remain strictly confidential.

## Results

**Figure 2:**
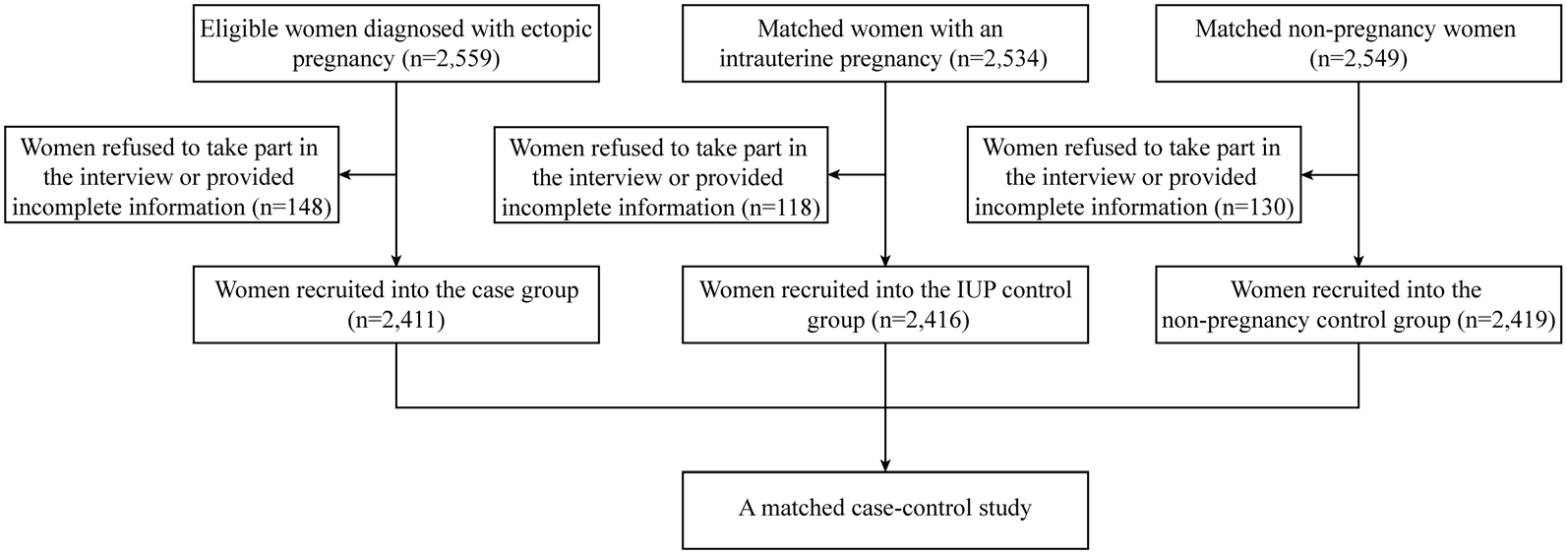
The recruitment profile for this study.

A total of 2,411 EP subjects, 2,416 matched IUP controls, and 2,419 matched non-pregnant controls were included in this study, after the exclusion of 148 EP patients, 118 IUP women, and 130 non-pregnant women who refused the interview and withdrew from the study or provided incomplete information. The response rate for the study was 94.18% (the recruitment profile is shown in Fig. 2).

The differences in sociodemographic characteristics are shown in Table 1. A higher proportion of EP subjects were born outside of Shanghai, were self-employed or unemployed, and had lower education levels compared with the IUP and non-pregnant controls. In addition, Table 2 indicates the history of reproduction and gynecological disease, and previous surgery between the three groups. The proportion of women with a previous EP, previous CT infection, history of infertility, or previous adnexal surgery or appendectomy was significantly greater in the EP group than the IUP and non-pregnant groups.

**Table 1:** Baseline characteristics (socio-demographic characteristics).

	EP		IUP		NonP		*P-value*
	n^a^	(%)	n^a^	(%)	n^a^	(%)	
**Age (years)**							0.16
≤20	24	(1.00)	32	(1.32)	22	(0.91)	
20–24	363	(15.06)	398	(16.47)	394	(16.29)	
25–29	753	(31.23)	772	(31.95)	718	(29.68)	
30–34	793	(32.89)	755	(31.25)	749	(30.96)	
35–39	332	(13.77)	322	(13.33)	368	(15.21)	
≥40	146	(6.06)	137	(5.67)	168	(6.95)	
**Marital status**							0.56
Married	2,067	(85.80)	2,088	(86.42)	2,101	(86.85)	
Unmarried	342	(15.20)	328	(13.58)	318	(13.15)	
**Birthplace**							<0.01
Shanghai	698	(28.95)	775	(32.08)	927	(38.32)	
Outside of Shanghai	1,713	(71.05)	1,641	(67.92)	1,492	(61.68)	
**Education attainment**							<0.01
College or above	1,061	(44.01)	1,378	(57.04)	1,256	(51.92)	
High school	314	(13.02)	280	(11.59)	275	(11.37)	
Middle school	178	(7.38)	195	(8.07)	188	(7.77)	
Primary school or lower	858	(35.59)	563	(23.30)	700	(28.94)	
**Occupation**							<0.01
Employed	1,682	(69.88)	1,897	(78.58)	1,928	(79.70)	
Self-employed	262	(10.88)	184	(7.62)	209	(8.64)	
Unemployed	463	(19.24)	333	(13.79)	282	(11.66)	
**Personal annual income (RMB)**							0.13
<50,000	1,165	(48.32)	1,093	(45.24)	1,111	(45.93)	
50,000–100,000	777	(32.23)	841	(34.81)	852	(35.22)	
>100,000	469	(19.45)	482	(19.95)	456	(18.85)	
**Smoking status** b							0.41
Nonsmoker	2,298	(95.31)	2,294	(96.18)	2,290	(96.10)	
Occasional smoker	63	(2.61)	57	(2.39)	57	(2.39)	
Regular smoker	50	(2.07)	34	(1.43)	36	(1.51)	
**Medical center** c							1.00
1	1,404	(58.23)	1,409	(58.32)	1,408	(58.21)	
2	272	(11.28)	272	(11.26)	276	(11.41)	
3	276	(11.45)	274	(11.34)	276	(11.41)	
4	291	(12.07)	293	(12.13)	293	(12.11)	
5	168	(6.97)	168	(6.95)	166	(6.86)	

EP, ectopic pregnancy; IUP, intrauterine pregnancy; NonP, nonpregnancy; LNG-EC, levonorgestrel emergency contraception.

a The sum does not necessarily equal the sample size for all variables because of missing data.

b Occasional smoker: cigarette smoking more than 4 times a week but an average of less than one cigarette per day. Regular smoker: smoking more than one cigarette per day continuously or over a 6-month period.

c Center 1 = International Peace Maternity and Child Health Hospital; Center 2 = Shanghai First People’s Hospital; Center 3 = Songjiang Central Hospital; Center 4 = Songjiang Maternity and Child Health Hospital; Center 5 = Minhang Central Hospital.

**Table 2:** Baseline characteristics (history of reproduction, gynecology, and surgery).

	EP		IUP		NonP		*P-value*
	n^a^	(%)	n^a^	(%)	n^a^	(%)	
**Reproductive history**							
**Number of previous abortions**							0.71
0	873	(36.88)	930	(38.49)	948	(39.34)	
1	763	(32.23)	756	(31.29)	749	(31.08)	
2	485	(20.49)	497	(20.57)	484	(20.08)	
≥3	246	(10.39)	233	(9.64)	229	(9.50)	
**Parity**							<0.01
0	1,143	(48.29)	1,280	(52.98)	639	(26.54)	
1	994	(41.99)	973	(40.27)	1,465	(60.84)	
≥2	230	(9.72)	163	(6.75)	304	(12.62)	
**Gynecologic history**							
**Previous ectopic pregnancy**							<0.01
No	2,093	(86.81)	2,363	(97.81)	2,356	(97.40)	
Yes	318	(13.19)	53	(2.19)	63	(2.60)	
**Serum ** ***Chlamydia trachomatis*** ** IgG test**							<0.01
Negative	1,648	(69.13)	2,099	(89.55)	2,181	(90.91)	
Positive	736	(30.87)	245	(10.45)	218	(9.09)	
**Previous infertility**							<0.01
No	2,005	(83.26)	2,286	(95.65)	2,325	(96.47)	
Yes	403	(16.74)	104	(4.35)	76	(3.15)	
**Previous cesarean section** a							0.79
No	691	(56.09)	624	(54.74)	988	(55.72)	
Yes	541	(43.91)	516	(45.26)	785	(44.28)	
**Surgical history**							
**Previous adnexal surgery**							<0.01
No	1,985	(82.33)	2,322	(96.19)	2,242	(92.84)	
Yes	426	(17.67)	92	(3.81)	173	(7.16)	
**Previous appendectomy**							0.01
No	2,303	(95.84)	2,346	(97.47)	2,331	(96.44)	
Yes	100	(4.16)	61	(2.53)	86	(3.56)	

EP, ectopic pregnancy; IUP, intrauterine pregnancy; NonP, nonpregnancy; LNG-EC, levonorgestrel emergency contraception.

a The number of women who had delivered a child was used as the denominator to calculate the percentage.

The results of the analyses of the association between previous contraceptive use and EP risk are shown in Table 3. After adjustment, the data showed that only previous IUD users had a slight risk of EP (AOR_1_ = 1.87, 95% CI: 1.48–2.37; AOR_2_ = 1.84, 95% CI: 1.49–2.27) compared with previous non-IUD users, and the EP risk increased with the duration of previous use (P_1_ for trend <10^−4^, P_2_ for trend <10^−4^). The ORs were not significant among women with previous IUD use for less than one year (AOR_1_ = 1.27, 95% CI: 0.61–2.67; AOR_2_ = 1.26, 95% CI: 0.63–2.54); however, the ORs tripled when IUDs had been previously used for more than 8 years (AOR_1_ = 3.68, 95% CI: 2.17–6.24; AOR_2_ = 2.38, 95% CI: 1.50–3.79). Additionally, the crude OR of EP among women using the calendar rhythm method was significantly lower than that of women who did not use this method (OR_1_ = 0.78, 95% CI: 0.68–0.90; OR_2_ = 0.76, 95% CI: 0.67–0.89), but this difference was not significant after adjustment (AOR_1_ = 0.93, 95% CI: 0.77–1.13; AOR_2_ = 0.95, 95% CI: 0.79–1.14). The crude ORs of EP among women who previously underwent tubal sterilization were 7.12 (95%: CI 1.62–31.35) and 7.07 (95% CI: 1.61–31.14). However, after adjustment, the data showed that a previous tubal sterilization did not seem to increase the risk of EP (AOR_1_ = 2.51, 95% CI: 0.47–13.51; AOR_2_ = 2.66, 95% CI: 0.53–13.21).

**Table 3:** The association between EP risk and contraceptive method used during the previous cycle.

	EP		IUP		NonP		OR_1_ [95% CI]	OR_2_ [95% CI]	AOR_1_ [95% CI]^b^	AOR_2_ [95% CI]^b^
	n^a^	(%)	n^a^	(%)	n^a^	(%)	EP vs. NonP	EP vs. IUP	EP vs. NonP	EP vs. IUP
**Condom**										
No	684	(28.42)	665	(27.57)	523	(21.66)	*Reference*	*Reference*	*Reference*	*Reference*
Yes	1,723	(71.58)	1,747	(72.43)	1,892	(78.34)	0.70 [0.61, 0.79]	0.96 [0.85, 1.09]	1.15 [0.97, 1.37]	1.19 [1.00, 1.39]
**Withdrawal method**										
No	2,106	(88.94)	2,112	(87.53)	2,110	(88.32)	*Reference*	*Reference*	*Reference*	*Reference*
Yes	262	(11.06)	301	(12.47)	279	(11.68)	0.94 [0.79, 1.13]	0.87 [0.73, 1.04]	1.17 [0.90, 1.53]	1.13 [0.90, 1.40]
**Calendar rhythm method**										
No	1,920	(82.16)	1,933	(81.25)	1,879	(79.42)	*Reference*	*Reference*	*Reference*	*Reference*
Yes	417	(17.84)	446	(18.75)	487	(20.58)	0.78 [0.68, 0.90]	0.76 [0.67, 0.89]	0.93 [0.77, 1.13]	0.95 [0.79, 1.14]
**OCPs**										
No	2,254	(94.47)	2,295	(95.55)	2,271	(94.08)	*Reference*	*Reference*	*Reference*	*Reference*
Yes	132	(5.53)	107	(4.45)	143	(5.92)	0.93 [0.73, 1.19]	1.26 [0.97, 1.63]	0.94 [0.68, 1.31]	1.17 [0.86, 1.58]
**LNG-EC**										
No	1,283	(53.57)	1,311	(54.74)	1,330	(55.12)	*Reference*	*Reference*	*Reference*	*Reference*
Yes	1,112	(46.43)	1,084	(45.26)	1,083	(44.88)	0.94 [0.84, 1.05]	0.95 [0.85, 1.07]	1.06 [0.91, 1.24]	0.97 [0.84, 1.11]
**Tubal sterilization**										
No	2,374	(99.41)	2,402	(99.92)	2,417	(99.92)	*Reference*	*Reference*	*Reference*	*Reference*
Yes	14	(0.59)	2	(0.08)	2	(0.08)	7.12 [1.62, 31.35]	7.07 [1.61, 31.14]	2.51 [0.47, 13.51]	2.66 [0.53, 13.21]
**IUD^c^**										
No	1,993	(83.74)	2,130	(88.38)	2,110	(87.37)	*Reference*	*Reference*	*Reference*	*Reference*
Yes	387	(16.26)	280	(11.62)	305	(12.63)	1.34 [1.14, 1.58]	1.48 [1.25, 1.74]	1.87 [1.48, 2.37]	1.84 [1.49, 2.27]
**Duration of previous IUD use (years)^d^**										
<1	19	(4.91)	19	(6.79)	26	(8.52)	0.77 [0.43, 1.40]	1.07 [0.56, 2.02]	1.27 [0.61, 2.67]	1.26 [0.63, 2.54]
1–2	106	(27.39)	91	(32.50)	88	(28.85)	1.28 [0.96, 1.70]	1.25 [0.96, 1.66]	1.56 [1.06, 2.29]	1.61 [1.16, 2.24]
3–5	93	(24.03)	69	(24.64)	72	(23.61)	1.57 [1.16, 2.13]	1.73 [1.27, 2.37]	1.49 [0.98, 2.25]	1.82 [1.27, 2.63]
6–8	107	(27.65)	66	(23.57)	83	(27.21)	1.19 [0.88, 1.61]	1.44 [1.05, 1.98]	2.18 [1.47, 3.24]	2.08 [1.45, 3.00]
>8	62	(16.02)	35	(12.50)	36	(11.80)	1.82 [1.20, 2.76]	1.89 [1.25, 2.88]	3.68 [2.17, 6.24]	2.38 [1.50, 3.79]
*P_trend_*							<10^−3^	<10^−4^	<10^−4^	<10^−4^

EP, ectopic pregnancy; IUP, intrauterine pregnancy; NonP, nonpregnancy; OR, odds ratio; AOR, adjusted odds ratio; CI, confidence interval; OCPs, oral contraceptive pills; LNG-EC, levonorgestrel emergency contraception; IUD, intrauterine device.

a The sum does not necessarily equal the sample size for all variables because of missing data.

b Odds ratio was adjusted for birthplace, education attainment, occupation, parity, previous ectopic pregnancy, serum *Chlamydia trachomatis* IgG test, previous infertility, previous adnexal surgery, and previous appendectomy.

Table 4 presents the results of the analyses of the association between current contraceptive use and EP risk, with the group of women who did not use any contraceptives as a reference. Current use of any type of contraceptive significantly reduced the risk of unwanted IUP pregnancy (data shown in S1 Table). Furthermore, the risk of EP was also reduced with the current use of most methods except for LNG-EC (condom: AOR_1_ = 0.05, 95% CI: 0.04–0.06; withdrawal method: AOR_1_ = 0.13, 95% CI: 0.09–0.19; calendar rhythm method: AOR_1_ = 0.66, 95% CI: 0.48–0.91; OCPs: AOR_1_ = 0.14, 95% CI: 0.07–0.26; IUD: AOR_1_ = 0.17, 95% CI: 0.13–0.22; tubal sterilization: AOR_1_ = 0.04, 95% CI: 0.02–0.08; LNG-EC: AOR_1_ = 1.06, 95% CI: 0.80–1.41). However, in the case of contraceptive failure, current use of OCPs and LNG-EC increased the EP risk to approximately 4 times that in women who did not use any contraceptive method (OCPs: AOR_2_ = 4.06, 95% CI: 1.64–10.07; LNG-EC: AOR_2_ = 4.87, 95% CI: 3.88–6.10). Furthermore, the risk of EP following contraceptive failure was 7.68-fold higher in women with tubal sterilization (95% CI: 1.69–34.80) and 21.08-fold higher in current IUD users (95% CI: 13.44–33.07). The risk of EP following contraceptive failure among current IUD users increased with the duration of IUD use (P_2_ for trend <10^−4^).

**Table 4:** The association between EP risk and contraceptive method used in the current cycle.

	EP		IUP		NonP		OR_1_ [95% CI]	OR_2_ [95% CI]	AOR_1_ [95% CI]^b^	AOR_2_ [95% CI]^b^
	n^a^	(%)	n^a^	(%)	n^a^	(%)	EP vs. NonP	EP vs. IUP	EP vs. NonP	EP vs. IUP
**Current contraception**										
No	1,337	(55.87)	1,585	(65.77)	272	(11.33)	*Reference*	*Reference*	*Reference*	*Reference*
Condom	217	(9.07)	307	(12.74)	1251	(52.13)	0.04 [0.03, 0.04]	0.84 [0.69, 1.01]	0.05 [0.04, 0.06]	1.22 [0.98, 1.51]
Withdrawal method	59	(2.47)	89	(3.69)	133	(5.54)	0.09 [0.07, 0.13]	0.78 [0.56, 1.10]	0.13 [0.09, 0.19]	1.40 [0.97, 2.00]
Calendar rhythm method	171	(7.15)	251	(10.41)	77	(3.21)	0.45 [0.34, 0.61]	0.79 [0.64, 0.97]	0.66 [0.48, 0.91]	1.23 [0.97, 1.55]
OCPs	16	(0.67)	7	(0.29)	34	(1.42)	0.10 [0.05, 0.18]	2.71 [1.11, 6.61]	0.14 [0.07, 0.26]	4.06 [1.64, 10.07]
LNG-EC	341	(14.25)	145	(6.02)	96	(4.00)	0.72 [0.56, 0.94]	2.79 [2.27, 3.43]	1.06 [0.80, 1.41]	4.87 [3.88, 6.10]
IUD	231	(9.65)	24	(1.00)	470	(19.58)	0.10 [0.08, 0.12]	11.41 [7.45, 17.48]	0.17 [0.13, 0.22]	21.08 [13.44, 33.07]
Tubal sterilization	21	(0.88)	2	(0.08)	67	(2.79)	0.06 [0.04, 0.11]	12.45 [2.91, 53.18]	0.04 [0.02, 0.08]	7.68 [1.69, 34.80]
**Duration of IUD use (years)**										
No	1,337	(85.27)	1,585	(98.51)	272	(36.66)	*Reference*	*Reference*	*Reference*	*Reference*
<1	6	(0.38)	2	(0.12)	36	(4.85)	0.03 [0.01, 0.08]	3.56 [0.72, 17.66]	0.03 [0.01, 0.07]	5.87 [1.15, 30.06]
1–2	47	(3.00)	7	(0.44)	119	(16.04)	0.08 [0.06, 0.12]	7.96 [3.59, 17.67]	0.07 [0.05, 0.12]	14.29 [6.27, 32.56]
3–5	56	(3.57)	6	(0.37)	119	(16.04)	0.10 [0.07, 0.14]	11.07 [4.75, 25.76]	0.09 [0.06, 0.15]	21.62 [9.07, 51.55]
6–8	56	(3.57)	5	(0.31)	106	(14.29)	0.11 [0.08, 0.15]	13.28 [5.30, 33.24]	0.12 [0.07, 0.19]	26.77 [10.48, 68.39]
>8	66	(4.21)	4	(0.25)	90	(12.13)	0.15 [0.11, 0.21]	19.56 [7.11, 53.81]	0.22 [0.13, 0.35]	40.94 [14.54, 115.31]
*P_trend_*							<10^−4^	<10^−4^	<10^−4^	<10^−4^

EP, ectopic pregnancy; IUP, intrauterine pregnancy; NonP, nonpregnancy; OR, odds ratio; AOR, adjusted odds ratio; CI, confidence interval; OCPs, oral contraceptive pills; LNG-EC, levonorgestrel emergency contraception; IUD, intrauterine device.

a The sum does not necessarily equal the sample size for all variables because of missing data.

b Odds ratio was adjusted for age, birthplace, current address, education attainment, occupation, parity, previous ectopic pregnancy, serum *Chlamydia trachomatis* IgG test, previous infertility, previous adnexal surgery, previous appendectomy, and previous use of contraceptives, including condoms, withdrawal method, calendar rhythm method, OCPs, LNG-EC, tubal sterilization, and IUD.

## Discussion

In 2012, Wang *et al.* conducted a survey of the trends of contraceptive use and determinants of contraceptive choice between 1980 and 2010 in China. The study showed that although the use of long-term contraceptive methods, such as IUDs and female sterilization, remains predominant in China, the overall composition of contraceptive use within China has changed since the mid-1990s; furthermore, a higher education level was reported to correlate with a higher probability of using short-term contraceptive methods [16]. Another study also showed great differences in contraceptive use across regions with differing levels of socioeconomic development. The study also indicated that women using short-term contraceptive methods were more likely to live in urban than in rural areas [4]. The findings of the present study indicate that a large proportion of women used short-term contraceptive methods, including LNG-EC and condoms, and a small proportion used long-term contraceptive methods, such as IUDs and female sterilization. This finding is most likely related to rapid urbanization, a relatively higher educational level in Shanghai, and the considerable proportion (approximately 13%) of young and unmarried sexually active women in Shanghai with unintended pregnancies in recent years [19], [20] (the sociodemographic characteristics of current users of each method are shown in S2 Table). Thus, the overall distribution of contraceptives used in our study differs from that reported by Wang *et al.* and Zheng *et al*. for women throughout China.

Some epidemiology studies have reported that previous use of barrier methods, including condoms, can reduce the risk of EP [21], [22] because of their protective effects against sexually transmitted infections, including *Chlamydia trachomatis* and *Neisseria gonorrhea*, which are high-risk factors for EP [23]–[25]. In our study, however, previous condom use did not significantly decrease the risk of EP. This finding may be attributable to the lack of continuing condom use among the Chinese female population. Previous condom use was defined as condom use at least once during the previous cycle; thus, some episodes of intercourse may have occurred without barrier protection, increasing the chance of contracting sexually transmitted infections.

The risk of EP among women with previous use of behavioral contraceptive methods, including withdrawal and the rhythm method, was similar to that reported by Karare *et al.* [21], and current use of behavioral methods did not decrease the risk of EP following contraceptive failure. According to the data from the 2002 National Survey of Family Growth, withdrawal was considered ineffective in preventing pregnancy, with a failure probability of 18% [26], [27]. Rhythm methods were also unreliable as contraceptives because many women do not keep track of their last menstrual cycle, some track it incorrectly, some have irregular cycles, and the day of ovulation can vary from one cycle to another [28], [29]. Therefore, the risk of EP following contraceptive failure may not be reduced because of the inefficiency of behavioral methods.

The present study indicated that previous use of OCPs did not increase the risk of EP, which was in accordance with both population- and hospital-based case-control studies [30], [31]. Consistent with several case-control studies, including a multinational study from the World Health Organization (WHO) [22], [32]–[34], current OCP use can protect against unwanted IUP and EP, but it can quadruple the risk of EP following contraceptive failure compared with the risk associated with the non-use of any contraceptives. Due to the over-the-counter availability of most OCPs in China, and self-reported information collected from subjects, it’s hard for us to obtain the types of OCPs they used for further analysis. This study also revealed that previous and current use of LNG-EC yielded similar results to those of OCPs. It was surprising to find that current use of LNG-EC showed an increased risk for EP following its failure, which is contrary to the findings on a systematic review by Cleland *et al.* [35]. Although several clinical trials included in the systemic review have indicated the high efficiency of LNG-EC for emergency contraception, most trials did not use the incidence of EP following LNG-EC failure as an endpoint [36]–[42]. Thus, it remains debatable whether current use of LNG-EC increases the EP incidence following contraception failure. Furthermore, two epidemiology studies reported a higher incidence of EP following LNG-EC failure compared with the EP incidence in the general female population (2.3% reported by Lo *et al.* [43] and 4.1% reported by Gainer *et al*. [44]). Previous studies demonstrated that progesterone has a suppressive effect on human tubal cilia beats and smooth muscle contraction [45], [46], and this effect has been regarded as the main cause of impaired embryo-tubal retention and implantation [25]. Some Chinese women failed to appropriately use LNG-EC (including repeated use of LNG-EC in the same cycle, further acts of intercourse following taking LNG-EC, and etc.), which led to contraceptive failure. Thus, we speculated that in the case of contraceptive failure, the increased risk of embryo-tubal implantation may be attributed to the slowing effects of progesterone on embryo-tubal transport.

According to the results of this study, previous IUD use can slightly elevate the risk of EP, but current use can decrease the risk of EP. However, in the case of contraceptive failure, IUD users have a high risk of EP. The results of this study were confirmed by a meta-analysis that included 16 case-control studies [47]. It has been believed that the main cause of elevated EP risk in previous IUD users may be pelvic infections, which could lead to an ectopic implantation [48]–[50]. In terms of current use, IUDs show effectiveness in preventing intrauterine pregnancy and have been recommended by ACOG [51], but they are less effective for preventing extra-uterine pregnancy. Therefore, the ACOG Practice Bulletin also notes that “if pregnancy does occur with an IUD in place, the pregnancy is more likely to be ectopic” [51]. In addition, with the increasing use of levonorgestrel intrauterine systems, recent reports describe several EP cases following the failure of that method [52], [53]. One study showed an EP incidence of approximately 50% following the failure of levonorgestrel intrauterine systems in situ [54]. In China, there were many types of IUD used in the past. Women with an IUD use for a longer time in this study were more likely to have used the older models. Therefore, it remains unclear whether the effect of duration of IUD use caused by the types of IUD or the duration of its use. However, information on the type of IUD was difficult to obtain for further study. Despite this limitation, physicians should still be aware of the possibility of an EP following contraceptive failure among women with a long time use of IUD.

The insignificant increased risk of EP among women who underwent tubal sterilization reversals may be caused by the small proportion of previous contraceptive users in this study. Some studies have reported a high incidence of EP, ranging from 3.8%–7.7%, among those who had undergone tubal reanastomosis [55]–[57]. This finding can be attributed to potentially relevant factors, including the type of previous sterilization procedure, the surgical reversal technique used, and the discrepancy in diameter between the proximal and distal tube [55], [56]. However, the high risk of EP following contraceptive failure in women who undergo tubal sterilization may be caused by the altered structure of the fallopian tube and impaired embryo-tubal transport [58], and these results are in agreement with the results of other case-control studies that included a pregnant control group [32], [59].

The present study has some limitations. Data collection for this study was based on the subjects’ self-reports; thus, it was difficult to obtain information on types of IUD, OCPs, and sterilization methods for further study. Furthermore, a history of PID in this study was reported by subjects themselves. Except for those who had definite medical records of PID diagnosis, subjects without definite medical records, signs and symptoms reported “no” or “unsure”. Although female genital tract could be infected polymicrobially, CT was still reported as a leading pathogen associated with PID. It was estimated that 80% to 90% of the women with genitourinary CT infection are asymptomatic or subclinical [60]. Thus, CT IgG antibody was chosen as an objective index to report the PID condition of each subject without recall bias. Selection bias is another most common one in the hospital-based case-control study. However, we conducted a multicenter study with five hospitals that covered the urban and rural areas of Shanghai, which enabled us to recruit a relatively good representation of the general female population to minimize the influence of bias.

## Conclusion

In this large, multicenter case-control study of women from Shanghai, China, we found that current use of most contraceptives reduced the risk of both IUP and EP; however, if the contraceptive method failed, the proportions of EP might be higher than those of non-users, especially among women with current use of OCPs, LNG-EC, IUD, and tubal sterilization. Previous IUD use was associated with an increased EP risk, even when the device had been removed. In general, physicians should pay attention to women with previous long-term use of IUDs, and those who experienced contraceptive failure with current use of OCPs, LNG-EC, IUDs, and tubal sterilization, and EP diagnosis should be differentiated among them.

## Supporting Information

S1 Table **The association between intrauterine pregnancy risk and contraceptive method used in the current cycle.**
(XLSX)

S2 Table **Sociodemographic characteristics of the current users of each contraceptive method.**
(XLSX)
